# Comparison of the effects of apprenticeship training by sandwich feedback and traditional methods on final-semester operating room technology students’ perioperative competence and performance: a randomized, controlled trial

**DOI:** 10.1186/s12909-024-05598-6

**Published:** 2024-05-27

**Authors:** Azam Hosseinpour, Morteza Nasiri, Fatemeh Keshmiri, Tayebeh Arabzadeh, Hossein Sharafi

**Affiliations:** 1 Department of Operating Room, School of Allied Medical Sciences, Qom University of Medical Sciences, Qom, Iran; 2 Department of Anesthesia, School of Allied Medical Sciences, Tehran University of Medical Sciences, Tehran, Iran; 3 Department of Medical Education, Education Development Center, Shahid Sadoughi University of Medical Sciences, Yazd, Iran; 4 Department of Operating Room, Behbahan Faculty of Medical Sciences, P.O. Box 6361796819, Behbahan, Iran; 5 Student Research Committee, Iran University of Medical Sciences, Tehran, Iran

**Keywords:** Clinical competence, Formative feedback, Medical education, Perioperative nursing, Psychomotor performance

## Abstract

**Background:**

Effective feedback is fundamental in clinical education, as it allows trainers to constantly diagnose the trainees’ condition, determine their weaknesses, and intervene at proper times. Recently, different feedback-based approaches have been introduced in clinical training; however, the effectiveness of such interventions still needs to be studied extensively, especially in the perioperative field. Therefore, this study sought to compare the effects of apprenticeship training using sandwich feedback and traditional methods on the perioperative competence and performance of Operating Room (OR) technology students.

**Methods:**

Thirty final-semester undergraduate OR technology students taking the apprenticeship courses were randomly allocated into experimental (*n* = 15) and control (*n* = 15) groups through the stratified randomization approach. The students in the experimental group experienced Feedback-Based Learning (FBL) using a sandwich model, and the students in the control group participated in Traditional-Based Training (TBT) in six five-hour sessions weekly for three consecutive weeks. All students completed the Persian version of the Perceived Perioperative Competence Scale-Revised (PPCS-R) on the first and last days of interventions. Also, a blinded rater completed a checklist to evaluate all students’ performance via Direct Observation of Procedural Skills (DOPS) on the last intervention day. Besides, the students in the FBL filled out a questionnaire regarding their attitude toward the implemented program.

**Results:**

The mean total score of the PPCS-R was significantly higher in the FBL than in the TBT on the last intervention day (*P* < 0.001). Additionally, the increase in mean change of PPCS-R total score from the first to last days was significantly more in the FBL (*P* < 0.001). Likewise, the FBL students had higher DOPS scores than the TBT ones (*P* < 0.001). Most FBL students also had a good attitude toward the implemented program (*n* = 8; 53.3%).

**Conclusion:**

Apprenticeship training using a sandwich feedback-based approach was superior to the traditional method for enhancing perioperative competence and performance of final-semester OR technology students. Additional studies are required to identify the sustainability of the findings.

**Supplementary Information:**

The online version contains supplementary material available at 10.1186/s12909-024-05598-6.

## Background

A substantial concern in perioperative training is how trainees develop their technical or procedural skills and improve their performance and the required competencies for safe perioperative care and treatment [1]. Currently, most perioperative training programs are presented through apprenticeship courses in the Operating Room (OR), where most trainees encounter a high stress level as they are subjected to time pressures by the OR staff, making them susceptible to clinical errors [2]. In addition, due to restrictions on perioperative trainees’ work hours and trainers’ time, OR trainees confront challenges concerning traditional apprenticeship methods, such as being evaluated by inappropriate assessment methods and a lack of receiving constructive feedback on their educational endeavors [3, 4].

The OR program in Iran, a new four‑year centralized program called “OR technology”, lacks a well-designed or evidence-based approach to apprenticeship training, which may result in trainees’ failure to obtain the required skills and competencies [5]. However, considering the ever-increasing expectations of quality care in Iran, the emphasis of OR training in this country has been on trainees’ procedural skills and qualifications [6]. Based on the Iranian OR curriculum, most apprenticeship courses are trained with traditional methods, in which a faculty member, clinical instructor, or nurse manager supervises trainees in an actual OR when they act as technologists in specialized and sub‑specialized surgeries in two roles of a scrub or circulating person [7]. Besides, assessing OR trainees’ perioperative skills and competencies is a demanding issue in Iranian OR clinical courses because it is usually limited to traditional methods concentrated on subjectivity in assessment [8]. Hence, the perioperative competencies and procedural skills evaluation also has been criticized in Iranian traditional perioperative apprenticeship training as it is commonly summative, arbitrary, or subjective, without providing appropriate, purposeful, and structured formative feedback [9]. Consequently, it is indispensable to change or complement the traditional perioperative apprenticeship methods with new practical and well-established approaches to cultivate the required perioperative competencies and performance of Iranian OR trainees. To this end, there is a growing interest in Iranian perioperative studies to establish active and productive clinical training methods for OR trainees [10–13].

One of the fundamental components of higher education is feedback, allowing trainers to constantly diagnose the trainees’ condition, determine their weaknesses, and intervene at proper times [14]. Also, formative feedback is a highly influential factor in improving trainees’ learning performance because it helps them to identify and correct their mistakes in the learning process and carry out assigned tasks more precisely [15]. On the contrary, in the summative feedback commonly provided in the traditional training methods, in which the trainers inspect the trainees’ status at the end of the semester, trainees can experience ever-increasing knowledge gaps until they relinquish their control [14].

Recently, numerous models and approaches have been used by clinical trainers to plan effective feedback encounters, including the SET-GO model, the One-Minute Preceptor, the Pendleton Rules, the R2C2 (Rapport/Reaction/Content/Coach), and the ALOBA (Agenda Led Outcome-based Analysis) [16]. Another type of the most commonly identified feedback is a sequence of positive-corrective-positive statements known as sandwich feedback [17]. In this approach, first represented by LeBaron and Jernick in 2000, one dose of critical/corrective feedback sandwiches between two doses of positive/reinforcement feedback such that the first and last statements are positive [18]. Sandwich feedback is a well-structured model that needs low levels of feedback-giving expertise by the trainer and low self-assessment and reflection skills by the trainee, making it useful in different feedback encounters [16].

Although feedback based on trainees’ perioperative performance seems to be associated with improving their performance, there is a paucity of research in this area worldwide [4]. Given this reason, and since proper feedback on students’ educational endeavors is often overlooked in ordinary apprenticeship training and evaluation in Iran’s OR program, we sought to compare the impacts of Feedback-Based Learning (FBL) presented based on the sandwich approach following the Direct Observation of Procedural Skills (DOPS) to the Traditional-Based Training (TBT) on improving perioperative competence and performance of Iranian OR technology students. We hypothesized that, after the interventions, the perioperative competence of students who experienced FBL would be higher than those who received TBT. Also, we assumed that the FBL arm’s perioperative performance would be better than the TBT arm at the end of the training. Finally, after participating in the FBL program, we deemed that students would have a moderate-to-good attitude toward FBL.

## Methods

### Study design

This was a quasi-experimental study with a parallel group design of 30 final-semester OR technology students enrolled during their field apprenticeship courses in the second semester of the academic year 2022–2023. The study was reported according to the Guideline for Reporting Evidence-based Practice Educational Interventions and Teaching (GREET) [19].

### Course background

The Iranian four-year Bachelor of Science (BS) program in OR technology comprises 130 units (i.e., 22 general, 74 basic sciences and core, 18 clinical training, and 16 field apprenticeship). Students start their clinical training in the second semester, running concurrently with theoretical courses until the end of the sixth semester. In the seventh and eighth semesters, students only present in the OR to pass their field apprenticeship courses, which is mandatory to reach a BS degree [7]. During the field apprenticeship program, students will act as OR technologists in specialized and sub-specialized surgeries in two roles, a circulating person and a scrub person, under the supervision of expert staff or faculty members. At the end of each semester of field apprenticeship, students’ procedural skills and competencies will be evaluated as subjective or objective [6].

### Participants and setting

The participants were all OR technology students who experienced the field apprenticeship courses in their last semester of an eight-semester BS program at two educational hospitals affiliated with Qom University of Medical Sciences, Qom, Iran. The students who were guests from other universities and those absent for more than three sessions during the three weeks of the intervention were excluded. Also, the students who had any experience of clinical working in the OR outside of their school program were withdrawn from the study.

The eligible students were recruited via the census. Of the 30 eligible students, all voluntarily accepted to partake in the study. Stratified randomization was used to divide students into TBT (*n* = 15) and FBL (*n* = 15). To this end, first, recruitment hospitals and gender were considered potential covariates, and four stratifications were generated (i.e., first hospital and male, first hospital and female, second hospital and male, second hospital and female). Then, each student was given a number from 1 to 30 and allocated to an appropriate combination of generated stratifications. Finally, students of each combination of stratifications were assigned equally to the study groups utilizing a random number software.

### Data collection tools

Data were collected with a demographic-educational information form, the Persian version of the Perceived Perioperative Competence Scale-Revised (PPCS-R), the checklist of DOPS for OR technology students, and the FBL attitude questionnaire. The first tool includes information on students’ age, gender, recruited hospital, and the previous semester’s grade point average; all documented before group allocation. The PPCS-R was filled out by all students on the first and last days of three-week interventions to measure their perioperative competence as pre-test and post-test. However, the DOPS checklist was completed only on the last day of the three-week intervention to assess students’ perioperative performance during DOPS. To control the rater’s bias, the DOPS checklist was completed for all students by a blinded rater with good experience in performing DOPS, who was unfamiliar with the recruited students and unaware of their assigned groups. The DOPS checklist was also fulfilled during DOPS by an OR instructor for the FBL students as a part of the feedback-based strategy. The FBL attitude questionnaire was also completed on the last day of the three-week intervention by FBL students to measure their attitude regarding the implemented program. Additionally, the FBL instructors’s perspectives regarding the implemented program were narrated to better understand the program’s value.

The PPCS-R is a self-completed tool developed by Mirbagher Ajorpaz et al. (2016) to assess the clinical competence of final-semester OR technology students in their apprenticeship courses. The scale contains 33 items on a five-point Likert scale from strongly disagree (score: 1) to strongly agree (score: 5). The scale also has five dimensions: (1) “foundational skills and knowledge” (seven items), (2) “leadership” (nine items), (3) “collegiality” (seven items), (4) “proficiency” (four items), and (5) “professional development” (six items). The scale total score, varying from 33 to 165, is computed by summing up the scores of five dimensions. A higher score demonstrates a higher level of clinical competence. A good fit to the data was reported in the first psychometric assessment of this scale in the Iranian context (goodness of fit index = 0.86, adjusted goodness of fit index = 0.90, comparative fit index = 0.90, normed fit index = 0.84, and root mean square error of approximation = 0.04). The internal consistency was also established by obtaining Cronbach’s alpha (*α*) of 0.86 for the entire scale and 0.62 to 0.70 for five dimensions [20]. In the current study, we obtained a Cronbach’s alpha of 0.74 for the entire scale, using data gathered in the pre-test.

The DOPS checklist for OR technology students was developed by Nayyeri et al. (2021) to evaluate the common technical-surgical skills of Iranian final-semester OR technology students via DOPS. The checklist includes the following eight domains (one question per domain), which could be assessed in all educational hospitals for all students: (1) “information about the anatomy of the surgical site”, (2) “communication with the patient”, (3) “pre-operative measures”, (4) “compliance with sterile conditions”, (5) “technical skills in surgery”, (6) “post-operative measures”, (7) “communication with the members of the surgical team”, and (8) “professional behavior”. Each question is scored on a five-point Likert scale, including unobservable (score: 1), less than expected (score: 2), borderline (score: 3), as expected (score: 4), and more than expected (score: 5). The checklist total score ranges from 8 to 40, and a higher score displays a better level of perioperative performance. Nayyeri et al. confirmed the validity and test-retest reliability of the DOPS checklist with a Content Validity Index (CVI) of 0.80 and the Intraclass Correlation Coefficient (ICC) of 0.93, respectively. They also reported good internal consistency with a Cronbach’s alpha of 0.90 [21]. We obtained a Cronbach’s alpha of 0.88 for internal consistency in the current study. Also, to establish the inter-rater reliability, 30 students with similar educational characteristics to those of the target population were evaluated by two raters, and the ICC coefficient for raters agreement was found to be 0.76.

The FBL attitude questionnaire was designed by Ghilay (2018) to examine students’ views towards the FBL model in higher education. This self-report questionnaire includes 19 items on a five-point Likert scale from strongly disagree (score: 1) to strongly agree (score: 5). The questionnaire consists of four dimensions: (1) “diagnosis” (i.e., identifying learning difficulties, four items, *α* = 0.88), (2) “prognosis” (i.e., management of problems, seven items, *α* = 0.94), (3) “motivation and sense of belonging” (four items, *α* = 0.80), and (4) “the contribution of FBL to learning improvement” (four items, *α* = 0.90). The total questionnaire score varies from 19 to 95, with higher obtained scores describing better attitudes [14].

The FBL attitude questionnaire in the current study was translated from English to Persian with a forward-backward technique while preserving its original content. Then, the validity and reliability of the Persian version were assessed. To address content validity, 12 medical education professionals with experience in testing the psychometry properties of educational tools were invited purposively. In the first stage, they investigated the questionnaire qualitatively regarding objectivity, the number of items, and the logical sequence of items. Subsequently, based on the experts’ feedback, the questionnaire was revised and sent back to them to evaluate the 19 modified items regarding quantitative content validity. The experts determined all items as relevant and essential, indicating a minimum Item-level Content Validity Index (I-CVI) of 0.97 and the Content Validity Ratio (CVR) of 0.88, which are satisfactory [22]. Besides, the qualitative face validity of the final Persian version was established in terms of difficulty, ambiguity, and syntax by 30 students with identical features to those of the target population, who were chosen purposively and not incorporated in the principal analysis. Also, the questionnaire’s internal consistency was reasonable, as it obtained 0.87 by Cronbach’s alpha for the entire tool. According to the statistical adviser, the total score of the questionnaire was categorized as weak (below the median, score < 65), moderate (between the median and the third quartile, score: 65–69), and good (above the third quartile, score > 69).

### Educational intervention

We applied identical lesson plans and evaluation techniques for students of TBT and FBL groups. Based on the recruiting university regulations, 15 students of each study group were divided into a group of seven students and eight students. Then, an OR instructor trained and supervised each group of seven to eight students in six five-hour field apprenticeship sessions weekly for three consecutive weeks. To reduce bias, each generated group of students was randomly assigned to the first and second hospitals with the same surgical procedures and environments. The study was performed first for eight students of the FBL group in the first hospital and eight students of the TBT group in the second hospital during the first three weeks of the semester. Then, it was conducted during the second three weeks of the semester for seven students of the FBL group in the second hospital and seven students of the TBT group in the first hospital.

Four eligible OR instructors were randomly assigned to the four generated groups of students to train and supervise the students (one per group). Only instructors with five years of experience in teaching the field apprenticeship courses were selected to reduce bias regarding using different instructors. Also, the program coordinator briefed all instructors regarding the program structure and process before initiating the study. The DOPS, performed to compare the students’ perioperative performance between the study groups, was also held under the same formats and conditions for the groups by a rater with prior experience in conducting the DOPS for OR students, who was unaware of the group assignment. To this end, on the last day of the three-week interventions, the rater observed students while doing skills and recorded their observations in the DOPS checklist. Eventually, the final score was noted for each student, which was only for research purposes. On the other hand, to report the student’s final score based on university regulations, an unstructured practical exam was held at the end of the semester, and students’ procedural skills and competencies were evaluated subjectively.

Before the commencement of the study, a briefing session was held for the two groups’ students regarding the research objectives, the type of desired technical-surgical skills, and their evaluation method. Then, students in the TBT group received traditional training, whereas those in the FBL group were taught with a feedback-based approach. The study was performed for two groups during the first and second three weeks of the semester. In the following weeks of the semester, students of two groups were taught with a traditional approach.

In the FBL group, during the first week, the corresponding instructor presented practical training on the desired technical-surgical skills and observed and evaluated the students’ perioperative performance using the DOPS checklist in 20–30 min. Immediately afterward, structured feedback was given to students in five to ten minutes in a private meeting by the corresponding instructor, using the sandwich method (i.e., highlighting both strengths and weaknesses of student performance). To this end, initially, the strengths and positive aspects of the student regarding her/his performance were mentioned. Then, constructive feedback was provided, and her/his weaknesses and negative points were discussed. Finally, once again, the strengths and positive points were reminded of the student, and the action plan was suggested. The student was also urged to engage in more practice and study to improve her/his performance. Ultimately, the feedback summary was documented descriptively within each student’s portfolio. This process of evaluation and providing feedback was repeated one week after the first week. Finally, the student’s checklist scores in two evaluation stages were compared to assess the student’s progress in the desired skills. An overview of the implemented program for one student is presented in Supplementary 1.

According to the traditional apprenticeship program, an OR instructor taught the desired skills and asked the students to accomplish the skills independently and frequently. In this method, general feedback was also provided to a group of students at the end of an apprenticeship day or the beginning of the following apprenticeship day. Additionally, individual feedback was presented immediately in emergencies and based on demand, but it was provided to all students in a written evaluation form at the end of the semester. In the traditional program, both group and individual feedback were provided based on the instructor’s own principles in an unstructured and arbitrary format, without following any standard in terms of steps of feedback provision and frequency. In these cases, no standard follow-up was conducted after feedback to see if students were improving based on the presented feedback.

### Ethical considerations

The study was approved by the Regional Research Ethics Committee of Behbahan Faculty of Medical Sciences, Khozestan, Iran. Before the study commenced, all eligible students signed written informed consent after being provided with the research methods and objectives.

### Data analysis

All analyses were run with the Statistical Package for Social Sciences software (version 25.00; SPSS Inc., USA). The Kolmogorov-Smirnov test did not confirm the normal distribution of data. Accordingly, the homogeneity of study groups for demographic-educational information was addressed with the Chi-square and Mann-Whitney U tests. Also, to compare the means of PPCS-R score between groups at baseline and post-test, the Mann-Whitney U test was employed. Additionally, this test was utilized to compare the groups regarding the mean changes in PPCS-R score from the baseline to the post-test. Finally, we used the Mann-Whitney U test to compare the post-test score of the DOPS checklist between the study groups. The significance level was deemed less than 0.05 in all tests.

## Results

### Basic characteristics

All 30 randomized students ended the trial and were incorporated into the final analysis. The results revealed no significant difference between the two groups regarding demographic-educational data (Table 1).

**Table 1:** Basic characteristics of the final-semester operating room technology students in the study groups

Variables		FBL group^*^ (*n* = 15)	TBT group^**^ (*n* = 15)	*P*-value
**Gender**	**Female**	9 (60.0)	9 (60.0)	1.000^**†**^
	**Male**	6 (40.0)	6 (40.0)	
**Recruited hospitals**	**Shahid Beheshti**	8 (53.3)	7 (46.7)	1.000^**†**^
	**Nekouei-Hedayati-Forghani**	7 (46.7)	8 (53.3)	
**Age (years)**		22.73 ± 0.70	22.26 ± 0.70	0.098^**††**^
**Previous semester’s grade point average (range: 0–20)**		17.04 ± 1.14	17.28 ± 1.12	0.683^**††**^

Abbreviations: FBL, Feedback-Based Learning; TBT, Traditional-Based Training

All values have been expressed as mean ± standard deviation or number (percentage)

^*^ Received a sandwich FBL approach in six five-hour sessions weekly for three consecutive weeks

^**^ Received a TBT method in six five-hour sessions weekly for three consecutive weeks

^**†**^ Chi-square test

^**††**^ Mann-Whitney U test

### Perioperative competence

The students’ PPCS-R scores in the two study groups are presented in Table 2. The Mann-Whitney U test displayed no significant inter-group difference in the mean PPCS-R total score at baseline (*P* = 0.567). Yet, this test confirmed that the mean of the post-test total score obtained by the students in the FBL group was significantly higher than that acquired by the students in the TBT group (*P* < 0.001). Similarly, the mean scores of all PPCS-R dimensions were considerably higher in the FBL group than in the TBT group at the endpoint (*P* < 0.05). Also, the mean change of the PPCS-R total score from the baseline to the post-test was significantly higher in the FBL group compared to the TBT group (*P* < 0.001). Such finding was also found for the mean difference of all PPCS-R dimensions (*P* < 0.05), except for the “professional development dimension” (*P* = 0.285).

**Table 2:** The perioperative competence of the final-semester operating room technology students in the study groups

Variables (score)		FBL group^*^ (*n* = 15)	TBT group^**^ (*n* = 15)	*P-value* ^†^	Changes compared with pre-test			
					FBL group^*^	TBT group^**^	*P-value* ^†^	Effect size^††^
**Foundational skills and knowledge dimension (range: 7–35)**	Pre-test	13.40 ± 3.04	14.00 ± 1.81	0.367	-	-	-	
	Post-test	19.53 ± 2.03	17.60 ± 1.88	0.008	6.13 ± 3.27	3.60 ± 2.19	0.021	0.90
**Leadership dimension (range: 9–45)**	Pre-test	19.80 ± 1.74	20.46 ± 2.13	0.436	-	-	-	
	Post-test	28.73 ± 2.49	26.20 ± 2.21	0.006	8.93 ± 2.98	5.73 ± 3.08	0.009	1.05
**Collegiality dimension (range: 7–35)**	Pre-test	19.00 ± 1.85	19.66 ± 1.39	0.285	-	-	-	
	Post-test	24.53 ± 1.68	22.40 ± 1.50	0.001	5.53 ± 2.32	2.73 ± 1.98	0.002	1.29
**Proficiency dimension (range: 4–20)**	Pre-test	8.06 ± 1.22	8.80 ± 1.14	0.161	-	-	-	
	Post-test	12.60 ± 1.18	10.00 ± 0.92	< 0.001	4.53 ± 1.45	1.20 ± 1.61	< 0.001	2.17
**Professional development dimension (range: 6–30)**	Pre-test	16.60 ± 1.84	16.26 ± 1.75	0.576	-	-	-	
	Post-test	21.26 ± 1.33	19.73 ± 1.48	0.010	4.66 ± 2.41	3.46 ± 2.47	0.285	0.49
**Total perioperative competence (range: 33–165)**	Pre-test	76.86 ± 5.80	79.20 ± 4.36	0.567	-	-	-	
	Post-test	106.66 ± 4.40	95.93 ± 3.28	< 0.001	29.80 ± 4.53	16.73 ± 5.59	< 0.001	2.56

Abbreviations: FBL, Feedback-Based Learning; TBT, Traditional-Based Training

All values have been expressed as mean ± standard deviation

^*^ Received a sandwich FBL approach in six five-hour sessions weekly for three consecutive weeks

^**^ Received a TBT method in six five-hour sessions weekly for three consecutive weeks

^**†**^ Between-group *P-value*: Mann-Whitney U test

^**††**^ Cohen’s d

### Perioperative performance

As demonstrated in Table 3, the Mann-Whitney U test showed that the mean scores of the DOPS checklist recorded for the students in the FBL group were significantly higher than those recorded for the students in the TBT group (*P* < 0.001).

**Table 3:** The perioperative performance of the final-semester operating room technology students in the study groups

Variables (score)	FBL group^*^ (*n* = 15)	TBT group^**^ (*n* = 15)	*P*-value^†^	Effect size^††^
**Information about the anatomy of the surgical site (range: 1–5)**	3.80 ± 0.41	2.40 ± 0.50	< 0.001	3.06
**Communication with the patient (range: 1–5)**	4.06 ± 0.59	2.66 ± 0.48	< 0.001	2.60
**Pre-operative measures (range: 1–5)**	4.26 ± 0.45	2.73 ± 0.59	< 0.001	2.91
**Compliance with sterile conditions (range: 1–5)**	4.06 ± 0.25	2.60 ± 0.50	< 0.001	3.69
**Technical skills in surgery (range: 1–5)**	3.60 ± 0.50	2.33 ± 0.48	< 0.001	2.59
**Post-operative measures (range: 1–5)**	4.20 ± 0.56	2.66 ± 0.48	< 0.001	2.95
**Communication with the surgical team members (range: 1–5)**	4.20 ± 0.56	2.86 ± 0.35	< 0.001	2.86
**Professional behavior (range: 1–5)**	4.26 ± 0.59	2.60 ± 0.50	< 0.001	3.03
**Total perioperative performance (range: 8–40)**	32.46 ± 2.74	20.86 ± 2.64	< 0.001	4.31

Abbreviations: FBL, Feedback-Based Learning; TBT, Traditional-Based Training

All values have been expressed as mean ± standard deviation

^*^ Received a sandwich FBL approach in six five-hour sessions weekly for three consecutive weeks

^**^ Received a TBT method in six five-hour sessions weekly for three consecutive weeks

^**†**^ Between-group *P-value*: Mann-Whitney U test

^**††**^ Cohen’s d

### Attitude toward FBL

The mean scores of the FBL attitude questionnaire obtained by students of the FBL group are presented in Table 4. Students’ attitudes toward FBL were mostly good (*n* = 8; 53.3%) or moderate (*n* = 5; 33.3%).

**Table 4:** The attitude toward feedback-based learning among the final-semester operating room technology students of the feedback-based learning group (*n* = 15)

Variables (score)	Mean ± standard deviation
**Diagnosis dimension (range: 4–20)**	14.40 ± 2.06
**Prognosis dimension (range: 7–35)**	25.46 ± 2.19
**Motivation and sense of belonging dimension (range: 4–20)**	15.06 ± 1.90
**Contribution of feedback-based learning to learning improvement dimension (range: 4–20)**	15.33 ± 1.63
**Total attitude toward feedback-based learning (range: 19–95)**	70.26 ± 6.29

### Instructors’ perspectives

The FBL instructors were satisfied with the implemented program, and they felt that it could easily be incorporated into the daily routine by allocating enough training time and following a well-established approach. They also perceived that the presented feedback helped students to improve their knowledge and skills, as students thought of an idea as to whether the knowledge possessed was proper and relevant to their learning objectives and whether their performance was up to expected standards. Also, from the instructors’ perspectives, the FBL method could help students to engage in self-evaluation as well as boost their feedback-seeking behaviors and self-development concepts (i.e., self-motivation, self-confidence, self-esteem, and a sense of personal satisfaction), evoke their emotional reactions, and build a good relationship between students and their instructors, students and OR staff, students and patients, as well. The following are examples of quotes from the instructors:

If I did not provide feedback when I was busy, some students asked me eagerly to talk about their performance.

When I presented positive feedback regularly, it was followed by positive student reactions, and vice versa.

## Discussion

This study sought to compare the impacts of apprenticeship training by sandwich feedback and traditional methods on perioperative competence and performance of final-semester OR technology students in an Iranian population. The results revealed that the students who received sandwich feedback following the DOPS assessment gained higher perioperative competence scores than those who trained by the traditional method without receiving specific or structured feedback. Also, the mean change of perioperative competence compared to the baseline was significant after training among students who received the sandwich feedback approach compared to those who received the traditional method. In addition, the students in the sandwich feedback group obtained higher DOPS scores than those in the traditional group, suggesting their better perioperative performance. Thus, the results backed the assumption that the sandwich feedback method could enhance perioperative competence and performance among OR technology students. Also, this method could improve students’ attitudes toward FBL.

The current study findings substantiate the evidence that evaluated the effectiveness of novel apprenticeship training approaches in the competence of OR students in Iran. In a recent study, Bahadori et al. indicated that final-semester OR technology students’ PPCS-R scores were significantly higher in the task-based learning group than in the mentorship group [13]. Also, Mirbagher Ajorpaz et al. documented that OR apprenticeship training by mentorship, compared to traditional training, was associated with a significant increase in scores of PPCS-R [5]. Similarly, Sharif et al. reported that OR students’ scores on PPCS-R increased more after receiving the mastery learning model than the traditional method [23]. The results of the present study are compatible with the literature mentioned above, which showed the superiority of active-learning approaches compared to traditional ones in improving the students’ perioperative competence. The effectiveness of the sandwich feedback approach on the perceived perioperative competence in the current study can be justified by the fact that following proper and effective feedback, the student’s awareness of the developing competence will increase, resulting in a better evaluation of their clinical competence.

The current study also demonstrated that providing sandwich feedback following the DOPS assessment could enhance perioperative performance. Based on the results, two domains of “information about the anatomy of the surgical site” and “technical skills in surgery” had the lowest scores in both the FBL and TBT groups. According to the literature, limited investigations have been conducted to record feedback effects on the psychomotor skills of students in the OR fields. A recent systematic review reported that feedback based on intraoperative surgical performance was a powerful technique for improving the performance of surgical residents [4]. Also, Nayyeri et al. showed that using the DOPS evaluation accompanied by providing simple feedback compared to the traditional method substantially improved the perioperative performance of Iranian OR technology students [21]. The effectiveness of feedback on performance can be attributed to most students’ acceptance of this method, as feedback becomes valid and impactful when embraced by students [17].

Based on the findings, FBL could improve students’ attitudes toward FBL in four dimensions of “diagnosis”, “prognosis”, “motivation and sense of belonging”, and “the contribution of FBL to learning improvement”. Students’ perceptions indicated that FBL allowed instructors to make an adequate ongoing diagnosis and regularly understand their difficulties, weaknesses, and strengths; subsequently, the instructors mapped their problems, successfully dealt with them, and resolved them for each student. Also, based on students’ perceptions, FBL gave them the sense that the instructor was interested in them and their learning, which boosted their inspiration to learn and ask questions. Besides, the students believed that FBL substantially enhanced their learning process due to a better understanding of the material and meaningful learning. Previous studies in Iran also reported students’ positive attitudes toward receiving feedback, which agrees with the current study’s findings [15, 24]. In addition, we understood that the satisfaction of FBL instructors toward the implemented program was reasonable, and they believed that such programs are feasible to be incorporated into conventional training.

### Study strengths

This work is the initial attempt to implement sandwich feedback following the DOPS assessment. Besides, it is the second one regarding the use of DOPS assessment in Iranian OR technology students. We executed group allocation based on the stratified randomization method to reduce selection bias, which could be one of the most substantial aspects of this study. Additionally, to limit the dissimilarities in academic and environmental characteristics between groups, students were chosen from one university, and training was presented by the instructors with the same experience. Also, the DOPS rater was the same for all students and was blinded to group allocation, which could be another strength of the current study. Likewise, most previous related studies assess either competence or performance. However, we considered the evaluation of both perioperative competence and performance. Finally, we investigated students’ attitudes toward FBL and instructors’s perspectives regarding the implemented program.

### Study limitations

Although the study participants were recruited via census, the small sample size can limit the generalizability of the present findings. Likewise, the students were selected from a single center in Iran; therefore, the results might only be representative of some of the community. Finally, we performed a short-term intervention; hence, whether the observed effects persisted over a long period is unclear.

### Study implications for educational practice and future research

The current investigation could present beneficial evidence for OR instructors seeking innovative solutions to improve students’ perioperative competence and performance. Based on the results, providing sandwich feedback following the DOPS assessment can be potentially valuable as an alternative method to traditional approaches for apprenticeship training of Iranian OR technology students. Subsequently, since clinical instructors have an ethical and professional responsibility to foster students’ clinical competence and performance by implementing practical, easy usage, and evidence-based strategies, they are suggested to consider the FBL in a similar format in other schools. Nevertheless, to assess the generalizability of the findings and provide more reliable evidence on the efficacy of FBL in the perioperative field, forthcoming multi-center investigations with more samples are needed to consider the long-term impacts of this method on the study outcomes among students with comparable demographics or those who participate in other apprenticeship courses.

## Conclusions

Short-term FBL, which included providing sandwich feedback following the DOPS assessment for three consecutive weeks, was more effective than the traditional training in improving perioperative competence and performance of final-semester OR technology students. Also, the attitude toward FBL was good-to-moderate among the students who received the FBL. Similarly, the instructor’s satisfaction with the FBL was acceptable, and they believed that such programs are feasible to be integrated into conventional training.

### Electronic supplementary material

Below is the link to the electronic supplementary material.

Supplementary Material 1

Supplementary Material 2

## Data Availability

Data and materials are obtainable by contacting the corresponding author.

## Abbreviations

*a*: Cronbach’s alpha
ALOBA: Agenda Led Outcome-based Analysis
BS: Bachelor of Science
CVI: Content Validity Index
I-CVI: Item-level Content Validity Index
CVR: Content Validity Ratio
DOPS: Direct Observation of Procedural Skills
FBL: Feedback-Based Learning
GREET: Guideline for Reporting Evidence-based Practice Educational Interventions and Teaching
ICC: Intraclass Correlation Coefficient
OR: Operating Room
PPCS-R: Perceived Perioperative Competence Scale-Revised
R2C2: Rapport/Reaction/Content/Coach
SPSS: Statistical Package for Social Sciences software
TBT: Traditional-Based Training
