# Supplementary material for: Comparison of the effects of apprenticeship training by sandwich feedback and traditional methods on final-semester operating room technology students’ perioperative competence and performance: a randomized, controlled trial
Source: BMC Med Educ. 2024 May 27;24:578. doi: 10.1186/s12909-024-05598-6 (PMC11129480; doi:10.1186/s12909-024-05598-6)
Supplement: Supplementary file 2 — Supplementary Material 2 [file 12909_2024_5598_MOESM2_ESM.docx]

**Supplementary 1.** A summary of feedback was presented to one student of the feedback-based training group after appendectomy surgery.

| **Time** | **Outline** | **Strengths/Weaknesses** | **Action plan** |
| --- | --- | --- | --- |
| **First week** | - Mrs. SR, I saw you had a good relationship with the patient when the patient entered the surgery room. You were almost active in preparing the surgery room for appendectomy surgery, and you also observed all relevant points in preparing the electrocautery and cooperated with the staff actively. | - Strengths (score: 3, borderline): communication with the patient, pre-operative measures | - The instructor explained the importance of proper communication with the surgical team members in patient treatment. - The student was urged to study a guideline related to sterile conditions and asked to act as a scrub person in the following surgeries in the instructor's presence. - The instructor opened a sterile appendectomy instrument set and explained the instruments' names and uses to the student again. The instructor also explained the appendectomy steps and the anatomy related to the layers of the abdomen using an instructional video. The student was also asked to attend another appendectomy in the following days to enhance her learning and was re-evaluated by the instructor. |
|  | - On the other hand, I observed that you did not communicate effectively and appropriately with other surgical team members. You did not follow the sterile conditions when you scrubbed and opened the surgical sterile packs. Also, during the surgery, you did not have sufficient knowledge of the names of the surgical instruments and their timely use. These were less than I expected. | - Weaknesses (score: 2, less than expected): communication with the surgical team members, compliance with sterile conditions, technical skills in surgery |  |
|  | - Mrs. SR, you had almost correct information about the patient's anatomy. When the surgeon asked about the type of surgical incision, I saw that you answered; however, you did not know enough about the names of the opened layers of the abdomen. Also, your performance in suturing and dressing the surgical site was almost correct, and you followed the ethical principles regarding the patient's privacy. | - Strengths (score: 3, borderline): information about the anatomy of the surgical site, post-operative measures, professional behavior |  |
| **Second week** | - Mrs. SR, you made significant progress compared to the previous week. You prepared all the surgery equipment yourself. When the patient entered the surgery room, you had a good relationship with the patient. You asked the patient's name, introduced yourself, and increased your knowledge of abdominal anatomy. Your performance has improved as expected, and that's great. | - Strengths (score: 4, expected): pre-operative measures, communication with the patient, information about the anatomy of the surgical site | - The instructor explained the importance of professional behaviors in the operating room and their consequences for a person's professional performance through scenarios and cases. The student was also asked to discuss the moral cases she observed in the next session. - The student was asked to use surgical video animations on YouTube to master the surgical procedure. She was told that she would act as a scrub person in the subsequent surgery, and the instructor would re-evaluate her to check her mastery of the surgical process. |
|  | - Mrs. SR, I saw that you observed the aseptic and sterility techniques more than last week; your mastery of surgical instruments and their setup in the surgical table increased compared to the previous week. You also communicated more appropriately with the personnel than the last week. However, regarding professional behavior, you need to study more because you get a score of 3 out of 5 at this stage. Hence, it would help if you made more effort to progress further and reach the expected level in this domain. | - Strengths (score: 3, borderline): compliance with sterile conditions, technical skills in surgery, communication with the surgical team members - Weaknesses (score: 3, borderline): professional behavior |  |
|  | - Mrs. SR, your post-operative measures have progressed, especially in dressing and skin suturing, and you have received an acceptable grade within the expected range. | - Strengths (score: 4, expected): post-operative measures |  |
| **Third week (final DOPS)** | - After the final evaluation, Mrs. SR, you made significant progress in communicating with the surgical team members, but your most minor progress was in your surgical technical skills. | - Strengths (score: 5, more than expected): communication with the patient, post-operative measures, communication with the members of the surgical team, professional behavior - Weaknesses (score: 4, expected): information about the anatomy of the surgical site, pre-operative measures, compliance with sterile conditions - Weaknesses (score: 3, borderline): technical skills in surgery |  |
